# Acute Coronary Syndromes and SARS-CoV-2 Infection: Results From an Observational Multicenter Registry During the Second Pandemic Spread in Lombardy

**DOI:** 10.3389/fcvm.2022.912815

**Published:** 2022-06-16

**Authors:** Marco Ferlini, Diego Castini, Giulia Ferrante, Giancarlo Marenzi, Matteo Montorfano, Stefano Savonitto, Maurizio D’Urbano, Corrado Lettieri, Claudio Cuccia, Marcello Marino, Luigi Oltrona Visconti, Stefano Carugo

**Affiliations:** ^1^Division of Cardiology, Fondazione IRCCS Policlinico San Matteo, Pavia, Italy; ^2^Cardiology Department, ASST Santi Paolo e Carlo, Milan, Italy; ^3^Department of Clinical Sciences and Community Health, Division of Cardiology, University of Milan, Fondazione IRCCS Cà Granda Ospedale Maggiore Policlinico, Milan, Italy; ^4^IRCCS Centro Cardiologico Monzino, University of Milan, Milan, Italy; ^5^Interventional Cardiology Unit, IRCCS San Raffaele, Milan, Italy; ^6^Cardiology Department, Manzoni Hospital, Lecco, Italy; ^7^Cardiology Department, Legnano Hospital, ASST Ovest Milanese, Legnano, Italy; ^8^Cardiology Department, Carlo Poma Hospital, ASST Mantova, Mantua, Italy; ^9^Cardiology Department, Poliambulanza Hospital, Brescia, Italy; ^10^Cardiology Department, Ospedale Maggiore di Crema, ASST Crema, Crema, Italy

**Keywords:** acute coronary syndrome, COVID-19, coronary angiography, hub, STEMI (myocardial infarction)

## Abstract

**Background:**

COVID-19 had an adverse impact on the management and outcome of acute coronary syndromes (ACS), but most available data refer to March-April 2020.

**Aim:**

This study aims to investigate the clinical characteristics, time of treatment, and clinical outcome of patients at hospitals serving as macro-hubs during the second pandemic wave of SARS-CoV-2 (November 2020-January 2021).

**Methods and Results:**

Nine out of thirteen “macro-hubs” agreed to participate in the registry with a total of 941 patients included. The median age was 67 years (IQR 58-77) and ST-elevation myocardial infarction (STEMI) was the clinical presentation in 54% of cases. Almost all patients (97%) underwent coronary angiography, with more than 60% of patients transported to a macro-hub by the Emergency Medical Service (EMS). In the whole population of STEMI patients, the median time from symptom onset to First Medical Contact (FMC) was 64 min (IQR 30-180). The median time from FMC to CathLab was 69 min (IQR 39-105). A total of 59 patients (6.3%) presented a concomitant confirmed SARS-CoV-2 infection, and pneumonia was present in 42.4% of these cases. No significant differences were found between STEMI patients with and without SARS-CoV-2 infection in treatment time intervals. Patients with concomitant SARS-CoV-2 infection had a significantly higher in-hospital mortality compared to those without (16.9% vs. 3.6%, *P* < 0.0001). However, post-discharge mortality was similar to 6-month mortality (4.2% vs. 4.1%, *P* = 0.98). In the multivariate analysis, SARS-CoV-2 infection did not show an independent association with in-hospital mortality, whereas pneumonia had higher mortality (OR 5.65, *P* = 0.05).

**Conclusion:**

During the second wave of SARS-CoV-2 infection, almost all patients with ACS received coronary angiography for STEMI with an acceptable time delay. Patients with concomitant infection presented a lower in-hospital survival with no difference in post-discharge mortality; infection by itself was not an independent predictor of mortality but pneumonia was.

## Introduction

From the beginning of 2020, the world has had to face the COVID-19 pandemic caused by Severe Acute Respiratory Syndrome Coronavirus-2 (SARS-CoV-2) infection. Italy has been one of the most affected countries in Europe with more than seven million infections and over one hundred thousand deaths (1). In addition to mortality directly caused by severe acute respiratory syndrome and viral interstitial pneumonia, the COVID-19 pandemic played an indirect adverse effect on overall mortality excess, mainly by the necessity to divert resources from the optimal treatment of time-dependent medical and surgical emergencies to COVID-19 cases as a consequence of the dramatic surge in hospital admissions due to SARS-CoV-2 infection (2). An excess in cardiovascular deaths has been observed during 2020 compared to 2019 (3), which could be related to several factors, including reduction of acute coronary syndrome (ACS) hospitalizations, delay in ST-elevation myocardial infarction (STEMI) hospital presentation, an increase of out-of-hospital cardiac arrests, reduction of coronary revascularization procedures, and reduction of outpatient surveillance (4–7). Moreover, direct cardiac involvement has been reported in patients with COVID-19, and patients with ACS and concomitant infection had the worst outcome compared to patients without (8–12). Most of the available data refer to the first spread of the SARS-CoV-2 pandemic that occurred during the first months of 2020, while a second wave of the pandemic was observed worldwide between the end of 2020 and the beginning of 2021.

Lombardy, the most densely populated region in Italy, has been dramatically affected both during the first and the second wave of infection. To guarantee an optimal time of treatment for clinical emergencies, the regional healthcare authorities applied, during the first spread, a model of centralization called “macro-hubs” that was organized according to the estimated patient transportation time and the geographical features of the region. A detailed description of this model has been previously described and a retrospective analysis of its application, during the first wave, found an acceptable time delay in the ACS treatment (13, 14) of patients. This centralization model was, hence, further adopted during the second pandemic wave.

In the present study, we aimed to investigate the clinical characteristics, time to treatment, and clinical outcome of patients hospitalized at the macro-hub centers identified by the healthcare authorities of Lombardy during the second pandemic wave of SARS-CoV-2, from November 2020 to January 2021. Moreover, we performed an exploratory assessment of the GRACE score predictive performance in the present pandemic context.

## Materials and Methods

This study presents a retrospective analysis of prospectively collected data from a multicenter observational registry of consecutive patients with diagnoses of ACS hospitalized during the second SARS-CoV-2 pandemic spread. The macro-hubs involved in the registry and the duration of data collection (from 2 November 2020 to 31 January 2021) were based on the application of the decrees by Lombardy health authorities. The decrees defining a macro-hub were: (a) to perform primary percutaneous coronary intervention (PPCI) to all incoming STEMI on a 24/7 basis; (b) to guarantee a PPCI team was available 24/7 in the hospital (rather than on-call); (c) to provide separate pathways for patients with ACS and suspected/diagnosed COVID-19 from triage to catheterization laboratory and isolated care unit to avoid the risk of cross-infections.

At each participating hospital, a principal investigator was responsible for data collection in a custom electronic database provided by the coordinating center (Cardiology Department, University of Milan, ASST Santi Paolo e Carlo, Milan, Italy). At the end of data collection, the completed databases were submitted to the coordinating center for data analysis.

The study complies with the Declaration of Helsinki and was approved by the local institutional review board of each participating center. Patients gave their informed consent at admission for data collection and future publications in anonymous studies.

### Study Population

Eligible patients were included in the registry if they received a diagnosis of ACS during hospitalization. STEMI was defined as typical symptoms lasting at least 20 min and persistent ST-elevation of ≥ 2 mm in at least two contiguous leads or new or presumed new left bundle-branch block. NSTEMI was defined as new onset or worsening angina (or equivalent) and elevated biomarkers of myocardial necrosis (troponin I or T above the upper limits of normal at each study site) with or without associated electrocardiographic signs of ischemia (ST-depression, transient ST-elevation, or T-wave inversion). Unstable angina (UA) was defined by the absence of troponin elevation.

The diagnosis of SARS-CoV-2 infection was based on the positive nasopharyngeal swab, bronchoalveolar lavage, and a pulmonary TAC diagnostic for interstitial pneumonia, as a single test or in combination.

Patients with either STEMI or high-risk non-ST-elevation ACS (NSTE-ACS) (presence of hemodynamic and/or electrical instability, recurrent or ongoing chest pain refractory to medical treatments, and/or relevant ST-T wave changes) were directly transferred to the catheterization laboratory with the execution of a nasopharyngeal swab. Patients with low- or intermediate-risk NSTE-ACS were evaluated in the emergency department (ED) and underwent nasopharyngeal swab immediately, deferring percutaneous coronary intervention (PCI) decision after swab results and clinical conditions. All patients, regardless of the immediate treatment decision, were admitted to different wards according to their molecular nasopharyngeal swab results.

### Data Collection

For each patient, the following data were collected: demographic characteristics, cardiovascular risk factors, prior cardiac events or procedures, presence of cardiogenic shock, pulmonary edema or cardiac arrest on or before admission, site of STEMI at ECG, and echocardiographic left ventricular ejection fraction (LVEF). Moreover, blood hemoglobin, white blood cells, estimated glomerular filtration rate (eGFR) (CKD-EPI formula), and troponins values at admission were collected. Finally, the Global Registry of Acute Coronary Events (GRACE) score at admission was calculated (15). Data about in-hospital pharmacological treatments and interventional procedures had to be reported for all included patients.

For patients with STEMI, we analyzed the critical time intervals: “symptom-onset to first medical contact (FMC) (defined as the diagnosis by 12-lead electrocardiogram) and “FMC to arrival at catheterization laboratory (CathLab).”

As clinical adverse events, we considered the in-hospital occurrence of all-cause death, acute pulmonary edema, shock, cardiac arrest, acute kidney injury (AKI), major bleedings, pneumonia, and need for invasive and/or non-invasive ventilation. AKI was defined according to Kidney Disease Improving Global Outcomes (KDIGO) guidelines (16) and bleeding events were appraised according to Bleeding Academic Research Consortium (BARC) definitions (17). Total mortality was also collected at a 6-month follow-up.

### Statistical Analysis

Categorical data are reported as absolute values and percentages and compared using the chi-square test; continuous variables are described as the median and interquartile range (IQR) and compared using the Mann–Whitney test. The associations between clinical variables and clinical events were investigated using univariate and multivariate logistic regression analysis. The GRACE score predictive performance for in-hospital and post-discharge mortality was assessed using the C-statistic and receiver operating characteristic curves. The software used for statistical analysis was MedCalc Statistical Software version 16.2.0 (MedCalc Software bvba, Ostend, Belgium) and the cut-off adopted for statistical significance was *P* < 0.05.

## Results

Nine out of thirteen “macro-hubs” of the Lombardy region agreed to participate in the registry during the second pandemic wave and a total of 941 consecutive patients were included.

The baseline demographic and clinical characteristics and in-hospital treatments of the overall population are summarized in Table 1. The median age was 67 years (IQR 58-77), 30% were ≥ 75 years old, and 26% were females. STEMI was the clinical presentation in 54% of the cases (anterior site in 52%). The GRACE score at admission was 121 (IQR 100-143). Overall, 97% of the patients underwent coronary angiography (97.4% of STEMI and 96.8% of NSTE-ACS patients). Multivessel coronary artery disease (CAD) was present in 51% of cases, and there was no significant angiographic CAD in 8% of cases. A PCI was performed in 83.4% of the cases (90.7% of patients with STEMI and 74.8% of patients with NSTE-ACS), and coronary artery by-pass grafting (CABG) was performed in 6.5% of cases. Complete revascularization was obtained in 60% of cases within index admission.

**TABLE 1:** Baseline characteristics of the overall population.

VARIABLE	*N* = 941
Age, years, median (IQR)	67 (58–77)
Age ≥ 75 years, n (%)	284 (30)
Females, n (%)	242 (26)
Arterial hypertension, n (%)	625 (66.4)
Diabetes mellitus, n (%)	225 (24)
Hyperlipidemia, n (%)	477 (51)
Active smoking, n (%)	237 (25)
Previous MI, n (%)	195 (20.7)
Previous PCI, n (%)	212 (22.5)
Previous CABG, n (%)	54 (5.7)
**Clinical presentation**	
STEMI, n (%)	507 (54)
NSTE-ACS, n (%)	434 (46)
LVEF,%, median (IQR)	50 (40–55)
GRACE score, median (IQR)	121 (100–143)
Acute pulmonary edema, n (%)	55 (5.8)
Shock, n (%)	37 (3.9)
Cardiac arrest, n (%)	40 (4.3)
SARS-CoV-2 infection, n (%)	59 (6.3)
**Blood samples**	
Hemoglobin at admission, gr/dl, median (IQR)	14 (13–15)
White blood cells at admission, n/mcl, median (IQR)	9.8 (7.6–12)
Troponin at admission, ng/dl, medin (IQR)	0.25 (0.04–1.75)
eGFR at admission, ml/min/1.73 mq, median (IQR)	79.9 (59–92.6)
**Coronary angiography and revascularization**	
Coronary angiography, n (%)	914 (97)
STEMI, n (%)	494 (97.4)
NSTE-ACS, n (%)	420 (96.8)
Radial artery access, n (%)	809 (88.5)
PCI, n (%)	762 (83.4)
CABG, n (%)	60 (6.5)
Complete revascularization, n (%)	574 (60)
IABP, n (%)	56 (6)
PMCS, n (%)	7 (0.7)
**Drug therapy**	
Aspirin, n (%)	857 (91)
P2Y12 inhibitors, n (%)	778 (82.6)
Glycoprotein IIb/IIIa inhibitors, n (%)	125 (13.3)
Inotropic drugs, n (%)	91 (9.7)

*CABG, coronary artery by-pass grafting; eGFR, estimated glomerular filtration rate; IABP, intra-aortic balloon pump; LVEF, left ventricular ejection fraction; MI, myocardial infarction; NSTE-ACS, non ST elevation acute coronary syndrome; PCI, percutaneous coronary intervention; PMCS, percutaneous mechanic circulatory support; STEMI, ST elevation myocardial infarction.*

Sixty percent of the patients were transported to a macro-hub by the Emergency Medical Service (EMS), whereas 26% self-presented to the ED of a macro-hub and 12.8% were transferred from spoke centers; the remaining patients were already at the hospital at the time of ACS.

### Patients With Concomitant SARS-CoV-2 Infection

A total of 59 patients (6.3%) had concomitant confirmed SARS-CoV-2 infection. Table 2 shows the comparisons between demographic, baseline clinical characteristics, and in-hospital treatments of patients with and without SARS-CoV-2 infection.

**TABLE 2:** Comparison between patients with and without SARS-CoV-2 infection.

VARIABLE	SARS-CoV-2 (*N* = 59)	No SARS-CoV-2 (*N* = 882)	*P*-value
Age, years, median (IQR)	69 (62–77)	67 (58–77)	0.29
Age ≥ 75 years, n (%)	19 (32.2)	265 (30)	0.72
Females, n (%)	11 (18.6)	231 (26.2)	0.19
Arterial hypertension, n (%)	44 (74.6)	581 (65.9)	0.17
Diabetes mellitus, n (%)	19 (32.2)	206 (23.4)	0.12
Hyperlipidemia, n (%)	27 (45.8)	450 (51)	0.43
Previous MI, n (%)	16 (27)	179 (20.3)	0.21
Previous PCI, n (%)	16 (27)	196 (22.2)	0.38
**Clinical presentation**			
STEMI, n (%)	33 (56)	474 (53.7)	0.74
NSTE-ACS, n (%)	26 (44)	408 (46.3)	
LVEF,%, median (IQR)	48 (38–55)	50 (40–55)	0.09
GRACE score, median (IQR)	139 (105–158)	121 (100–142)	0.02
Acute pulmonary edema, n (%)	4 (6.8)	51 (5.8)	0.75
Shock, n (%)	3 (5.1)	34 (3.9)	0.64
Cardiac arrest, n (%)	3 (5.1)	37 (4.2)	0.74
Pneumonia, n (%)	25 (42.4)	7 (0.8)	<0.0001
**Blood samples**			
Hemoglobin at admission, gr/dl, median (IQR)	13.9 (12.3–15.4)	14 (12.8–15.2)	0.57
White blood cells at admission, n/mcl, median (IQR)	9.04 (7.55–11.19)	9.81 (7.64–12.20)	0.18
Troponin at admission, ng/dl, median (IQR)	0.61 (0.13–2.14)	0.24 (0.04–1.67)	0.04
eGFR at admission, ml/min/1.73 mq, median (IQR)	74 (52–90)	80 (59–93)	0.27
**Diagnostic and therapeutic procedures**			
Coronary angiography, n (%)	58 (98)	856 (97)	0.57
No significant CAD, n (%)	6 (10.3)	67 (8)	0.25
SVD, n (%)	18 (31)	355 (41.5)	
MVD, n (%)	34 (58.6)	434 (50.7)	
PCI, n (%)	47 (81)	715 (83.5)	0.62
CABG, n (%)	4 (6.9)	56 (6.5)	0.90
Complete revascularization, n (%)	29 (49)	545 (64)	0.04
IABP, n (%)	5 (8.5)	51 (5.8)	0.39
PMCS, n (%)	0 (0)	7 (0.8)	0.78
NIV, n (%)	13 (22)	26 (2.9)	<0.0001
IMV, n (%)	1 (1.7)	13 (1.5)	0.89
**Drug therapy**			
Aspirin, n (%)	52 (88)	805 (91)	0.41
P2Y12 inhibitors, n (%)	47 (79.7)	731 (82.9)	0.53
Glycoprotein IIb/IIIa inhibitors, n (%)	11 (18.6)	114 (12.9)	0.21
Inotropic drugs, n (%)	6 (10.2)	85 (9.6)	0.89

*CABG, coronary artery by-pass grafting; CAD, coronary artery disease; eGFR, estimated glomerula filtration rate; IABP, intra-aortic balloon pump; IMV, invasive mechanical ventilation; LVEF, left ventricular ejection fraction; MI, myocardial infarction; MVD, multivessel disease; NIV, non -invasive ventilation; NSTE-ACS, non ST elevation acute coronary syndrome; PCI, percutaneous coronary intervention; PMCS, percutaneous mechanic circulatory support; STEMI, ST elevation myocardial infarction; SVD, single vessel disease.*

In these patients, STEMI was the clinical presentation in 56% of cases (a rate comparable to that observed in patients without SARS-CoV-2 infection). The GRACE score was 139 (IQR 105-158), significantly higher than in patients without infection. Almost all patients (about 98%) underwent coronary angiography in both groups, and no significant differences were found in CAD extension; however, patients with SARS-CoV-2 infection presented a non-significant higher rate of no significant CAD (10.3 vs. 8%). PCI was performed in 81% of cases and CABG in 6.9%. Complete revascularization was obtained in 49% of cases, a significantly lower rate compared to that observed in patients without SARS-CoV-2 infection (64%, *P* = 0.04).

Pneumonia was present in 42.4% of patients with SARS-CoV-2 infection (vs.8% in patients without SARS-CoV-2 infection, *P* < 0.0001). Significantly more patients with COVID-19 underwent non-invasive ventilation (NIV) (22 vs. 2.9%, *P* < 0.0001), whereas no significant difference was observed regarding invasive mechanical ventilation utilization (IMV) between patients with and without COVID-19.

### Diagnosis and Treatment Times

Table 3 shows treatment times in the overall STEMI population and patients with and without SARS-CoV-2 infection.

**TABLE 3:** Time to treatment in the overall STEMI population and separately in patients with and without SARS-CoV-2 infection.

	Overall STEMI *N* = 507	SARS-Cov-2 *N* = 33	No SARS-Cov-2 *N* = 474	*P*-value
Symptom onset-FMC, median (IQR)	64 (30–180)	77 (37–240)	60 (30–180)	0.40
FMC-CathLab, median (IQR)	69 (39.5–105)	65 (37–160)	70 (40–125)	0.98

*FMC, first medical contact.*

In the whole population, the median time from symptoms-onset to FMC was 64 min (IQR 30-180). The median time from FMC to CathLab was 69 min (IQR 39-105). No significant differences were found between STEMI patients with and without infection in both time intervals.

### Clinical Outcomes

Table 4 summarizes the clinical outcomes observed in the overall population and separately in patients with and without SARS-CoV-2 infection.

**TABLE 4:** Clinical outcomes in the overall population and separately in patients with and without SARS-CoV-2 infection.

	Overall population	SARS-Cov-2	No SARS-Cov-2	*P*-value
Acute pulmonary edema, n (%)	38 (4)	1 (1.7)	37 (4.2)	0.34
Shock, n (%)	49 (5.1)	7 (11.9)	42 (4.8)	0.02
In-hospital cardiac arrest, n (%)	66 (7)	6 (10.2)	60 (6.8)	0.32
Major bleedings, n (%)	37 (3.9)	3 (5.1)	34 (3.8)	0.84
AKI, n (%)	91 (9.7)	10 (16.9)	81 (9.2)	0.13
In-hospital mortality, n (%)	42 (4.5)	10 (16.9)	32 (3.6)	<0.0001
Mortality at 6 months among hospital survivors, n (%)	36 (4.1)	2 (4.2)	34 (4.1)	0.98

*AKI, acute kidney injury.*

Except for cardiogenic shock, which was higher in patients with SARS-CoV-2 infection (11.9 vs. 4.8%, *P* = 0.02), no significant differences were found in the incidence of the other adverse events. In-hospital mortality was 4.5% in the overall population and was significantly higher in patients aged ≥ 75 years (8.1 vs. 2.9%, *P* = 0.004) and in STEMI (5.9 vs. 2.8%, *P* = 0.02).

In patients with concomitant SARS-CoV-2 infection, in-hospital mortality was significantly higher than in patients without (16.9 vs. 3.6%, *P* < 0.0001). Although in the univariate logistic regression analysis the presence of infection was significantly associated with in-hospital mortality (OR 5.41, 95% CI 2.51–11.65, *P* < 0.0001), in the multivariate analysis it showed a weak and not significant association, whereas the presence of pneumonia showed an independent association but with a borderline statistical significance (Table 5).

**TABLE 5:** Regression coefficients and odds ratios from multivariate logistic regression analysis testing association between clinical variables and in-hospital mortality.

VARIABLE	Regression coefficient (SE)	*P*-value	Odds ratios (95% CI)
Age	0.046 (0.024)	0.05	1.04 (0.99–1.09)
Diabetes mellitus	0.130 (0.542)	0.79	0.87 (0.30–2.52)
STEMI	0.445 (0.550)	0.41	1.56 (0.53–4.58)
MVD	1.237 (0.359)	0.02	3.44 (1.17–10.15)
LVEF ≤ 35%	1.568 (0.526)	0.003	4.79 (1.71–13.46)
eGFR < 60 ml/min/1.73 mq	1.027 (0.530)	0.05	2.79 (0.98–7.90)
Cardiac arrest	1.327 (1.160)	0.25	0.26 (0.02–2.57)
Shock	2.537 (0.670)	0.0002	12.65 (3.39–47.10)
SARS-CoV-2 infection	1.415 (0.834)	0.08	4.11 (0.80–21.12)
Pneumonia	1.732 (0.901)	0.05	5.65 (0.96–33.06)

*eGFR, estimated glomerular filtration rate; LVEF, left ventricular ejection fraction; MVD, multivessel disease; STEMI, ST elevation myocardial infarction. In the model were included all variables with P < 0.10 at the univariate analysis.*

Of the 899 patients discharged alive, mortality data at 6 months was available in 877 (98%). At this time point, mortality was 4.1% in the overall population and no significant difference was found between patients with and without SARS-CoV-2 infection (4.2 vs. 4.1%, *P* = 0.98). Infection was not significantly associated with post-discharge mortality. In the multivariate regression analysis only age, LVEF ≤ 35% at discharge, and the diagnosis of pneumonia were independently associated with post-discharge mortality.

In order to evaluate the predictive performance of the GRACE score in the present pandemic context, with particular regard to SARS-CoV-2 patients, we tested the predictive accuracy of the GRACE score at admission both for in-hospital and post-discharge mortality. Table 6 reports the results of the C-statistic. The score showed globally a good predictive performance for mortality, with higher C-statistic for in-hospital (0.85 95% CI.82–0.87, *p* < 0.0001) as compared to post-discharge mortality (0.75 95% CI.71–0.77, *p* < 0.0001), particularly with regard to in-hospital death in patients with concomitant SARS-CoV-2 infection (0.94 95% CI.82–0.98, *p* < 0.0001).

**TABLE 6:** Predictive values of the GRACE score for in-hospital and post-discharge mortality in the overall population and separately in patients with and without SARS-CoV-2 infection.

	C-statistic (95% CI)	Sens/Spec	*P*-value
**In-hospital mortality**			
Overall population	0.85 (0.82–0.87)	70/88	<0.0001
SARS-CoV-2 patients	0.94 (0.82–0.98)	100/88	<0.0001
NoSARS-CoV-2 patients	0.82 (0.79–0.85)	60/82	<0.0001
**Post-discharge mortality**			
Overall population	0.75 (0.71–0.77)	52/91	<0.0001
SARS-CoV-2 patients	0.82 (0.67–0.93)	100/62	<0.002
NoSARS-CoV-2 patients	0.73 (0.70–0.76)	50/90	<0.0001

## Discussion

In the present article, we describe the presentation, time of care, and mortality data of patients with ACS managed at hospitals identified as “macro-hubs” in a specific geographical area during the second spread of SARS-CoV-2 infection with a modified network of assistance based on a model of centralization of care.

The main findings of our analysis are as follows: more than half of patients presented with STEMI and these were treated within the ESC-recommended time delay (18); patients with ACS and positive at SARS-CoV-2 had a higher baseline risk profile, as suggested by a significantly higher GRACE score, and significantly higher mortality compared to patients without infection. This excess mortality risk appears to be attributable to the presence of concomitant pneumonia.

A delay in STEMI treatment was one of the first observations reported as a consequence of the COVID-19 outbreak at the beginning of the pandemic (19); particularly, patients with STEMI and COVID-19 presented the longest time of assistance as a consequence of a prolonged time from symptom onset to hospital admission, mainly due to the lack of dedicated organization of the healthcare system and for the limited availability of EMS due to systemic overload (12).

The centralized model used in Lombardy did not show a negative impact on time to treatment; furthermore, as previously reported, the time from symptom onset to CathLab was significantly shorter during the second compared to the first spread of infection (February-May 2020) (20). In the present analysis, about 60% of STEMI were directly transported to a macro-hub by EMS. The STEMI care network available for 15 years in the Lombardy Region comprising 55 CathLabs, mostly performing 24/7 primary PCI, and a well territorially distributed EMS certainly contributed to this positive result. However, the application of standardized protocols for fast-tracking the treatment of STEMI during the pandemic was endorsed by scientific societies, (21) allowing healthcare workers to obtain results in terms of the time of reperfusion, clinical outcomes, and staff safety in line with those before pandemic (22).

Patients with concomitant infection presented a significantly higher rate of in-hospital death compared to patients without infection (16.9 vs. 3.6%), whereas post-discharge mortality was not affected (4.2 vs. 4.1%); furthermore, in the multivariate analysis, infection by itself was not an independent predictor of mortality, whereas pneumonia was, though with a borderline statistical significance. It has been previously reported that patients with ACS, particularly STEMI, and concomitant COVID-19 present worse outcomes: in the North American COVID-19 Myocardial Infarction Registry, the in-hospital mortality of these patients was 33% (11). In the present data, a significant difference between patients with and without infection was found only in the rate of pneumonia and in the need of non-invasive ventilation: therefore, it is likely that pulmonary complications continue to have an adverse prognostic impact on these patients during the acute phase, whereas for survivors no significant difference in mortality was found at mid-term follow-up. However, we have reported a higher rate of pneumonia during the first spread of COVID-19 (about 60%) in patients with ACS and concomitant infection (14) that has been reduced (but not erased!) in the second wave by early and specific treatment (e.g., steroids and ventilation strategies); furthermore, the wide availability of diagnostic tools led to the diagnosis of patients with less severe clinical infection.

Although the GRACE score is a well-established predictive tool for outcomes prediction in patients with ACS (15), to our knowledge little information exists about its usefulness during the COVID-19 pandemic. Based on this, we tried an explorative investigation on the predictive value of the GRACE score on mortality and we found a good value of C-statistic for the overall population that was even stronger for patients with SARS-CoV-2 infection. Although the GRACE score is used to predict clinical outcomes in patients with ACS beyond infections, the baseline value was higher in patients with SARS-Cov2. These observations suggest that patients with ACS and SARS-CoV-2 might have a worse baseline risk profile and that the GRACE score retains a good predictive power in these patients. In a similar study, a significant difference was not found for GRACE score between patients with and without infection but a value > 140 and the presence of COVID-19 were independent risk factors associated with higher in-hospital mortality (23).

### Limitations

Small sample size, retrospective analysis, and lack of correction for covariates with consequent confounding bias can be considered as the main limitations of the present study. Furthermore, complete information on pharmacologic therapies was lacking. Finally, geographical differences do not allow definite conclusions and make our findings not necessarily representative of different areas in Italy or worldwide.

## Conclusion

The main aim of our work was to offer an overall clinical picture of ACS population during the second pandemic wave of SARS-CoV-2 infection and to describe its prognosis within the macro-hub network implemented by the Lombardy region in order to cope with the COVID-19 pandemic (13). The present article adds further confirmation to what we observed previously (14, 20): a timely adequate treatment of STEMI patients was obtained and a better prognosis in overall patients with ACS, both with and without SARS-CoV-2 infection, was observed during the second pandemic wave, corroborating in our opinion the beneficial effect of the organizational strategy adopted. Moreover, patients with concomitant infection had lower in-hospital survival, whereas post-discharge mortality was similar; infection by itself was not an independent predictor of mortality, whereas pneumonia implied a higher mortality risk.

## Data Availability Statement

The raw data supporting the conclusions of this article will be made available by the authors, without undue reservation.

## Author Contributions

SC, MF, and DC contributed to the conception and design of the study. DC, MF, and GF organized the database. DC performed the statistical analysis. MF and DC wrote the first draft of the manuscript. GM, MMo, SS, MD’U, CL, CC, MMa, and LV wrote the sections of the manuscript. All authors contributed to manuscript revision, read, and approved the submitted version.

## Conflict of Interest

The authors declare that the research was conducted in the absence of any commercial or financial relationships that could be construed as a potential conflict of interest.

## Publisher’s Note

All claims expressed in this article are solely those of the authors and do not necessarily represent those of their affiliated organizations, or those of the publisher, the editors and the reviewers. Any product that may be evaluated in this article, or claim that may be made by its manufacturer, is not guaranteed or endorsed by the publisher.
